# Clinical, Immunological, and Molecular Findings in Five Patients with Major Histocompatibility Complex Class II Deficiency from India

**DOI:** 10.3389/fimmu.2018.00188

**Published:** 2018-02-16

**Authors:** Jahnavi Aluri, Maya Gupta, Aparna Dalvi, Snehal Mhatre, Manasi Kulkarni, Gouri Hule, Mukesh Desai, Nitin Shah, Prasad Taur, Ramprasad Vedam, Manisha Madkaikar

**Affiliations:** ^1^Department of Pediatric Immunology and Leukocyte Biology, National Institute of Immunohaematology (ICMR), Mumbai, India; ^2^Division of Immunology, Bai Jerbai Wadia Hospital for Children, Mumbai, India; ^3^Pediatric Hematology-Oncology, P. D. Hinduja National Hospital & Research Center, Mumbai, India; ^4^Medgenome Labs Pvt. Ltd., Bangalore, India

**Keywords:** primary immunodeficiency disorder, flow cytometry, T cell receptor excision circles, next-generation sequencing, hematopoietic stem cell transplantation

## Abstract

Major histocompatibility complex (MHC) class II deficiency is a rare autosomal recessive form of primary immunodeficiency disorder (PID) characterized by the deficiency of MHC class II molecules. This deficiency affects the cellular and humoral immune response by impairing the development of CD4^+^ T helper (Th) cells and Th cell-dependent antibody production by B cells. Affected children typically present with severe respiratory and gastrointestinal tract infections. Hematopoietic stem cell transplantation (HSCT) is the only curative therapy available for treating these patients. This is the first report from India wherein we describe the clinical, immunological, and molecular findings in five patients with MHC class II deficiency. Our patients presented with recurrent lower respiratory tract infection as the most common clinical presentation within their first year of life and had a complete absence of human leukocyte antigen-antigen D-related (HLA-DR) expression on B cells and monocytes. Molecular characterization revealed novel mutations in *RFAXP, RFX5*, and *CIITA* genes. Despite genetic heterogeneity, these patients were clinically indistinguishable. Two patients underwent HSCT but had a poor survival outcome. Detectable level of T cell receptor excision circles (TRECs) were measured in our patients, highlighting that this form of PID may be missed by TREC-based newborn screening program for severe combined immunodeficiency.

## Introduction

Major histocompatibility complex (MHC) class II deficiency is a rare form of primary immunodeficiency disorder (PID) that follows an autosomal recessive pattern of inheritance. Worldwide, over 150 patients have been reported with this deficiency (1). About two-third of these patients have a North African descent (Algeria, Morocco, and Tunisia) (1) and belong to complementation group B. A high prevalence of group A deficiency is reported in the European countries (2). A defect in the factors that regulate the expression of MHC class II genes resulting in the absence or reduced expression of MHC class II molecules on the immune cells is thought to be the underlying cause of MHC class II deficiency (3). The MHC class II molecules also referred to as human leukocyte antigens (HLAs) are polygenic and highly polymorphic cell-surface glycoproteins that assemble as α and β chain heterodimers (3). Humans have three isotypes of HLA-class II molecules: HLA-DR, -DP, and -DQ, which are present on resting B cells, monocytes, dendritic cells, activated T lymphocytes, Langerhans cells, and epithelial cells in the thymus and intestine (4). Somatic fusion experiments supported the description of four complementation groups (A, B, C, and D) (1). Subsequently, four regulatory proteins were identified, deficiencies of which are associated with MHC class II deficiency: class II transactivator (CIITA) under group A (1), RFX containing ankyrin repeats (RFXANK) under group B (5), the fifth member of the RFX (RFX5) under group C (6), and RFX-associated protein (RFXAP) under group D (7). The RFXANK, RFX5, and RFXAP proteins are three subunits of the ubiquitously expressed RFX complex, which bind directly to the promoters of all MHC class II genes and associate with other pleiotropic factors to form the MHC class II enhanceosome (1).

The development and thymic shaping of CD4^+^ T helper (Th) cells require MHC class II-mediated antigen presentation to the T cell receptor (TCR) of CD4 cells. Hence, MHC class II deficiency affects both the cell-mediated and humoral immunity due to a defect in CD4^+^ Th cell development and lack of helper T cell-dependent antibody production by B cells. Affected children typically present with severe respiratory and gastrointestinal tract infections and are diagnosed within their first year of life. However, there are some reports with a milder clinical presentation and diagnosis later up to 15 years of age (3). Diagnosis involves an immunophenotypic finding of a reduced absolute CD4^+^ Th count, inverse CD4:CD8 ratio, an absent or reduced expression of HLA-DR on lymphocytes and monocytes, hypogammaglobulinemia, and a genetic defect in any of the four genes producing the regulatory proteins.

Allogeneic hematopoietic stem cell transplantation (HSCT), preferably from an HLA-identical sibling, is currently the only available curative treatment for this disorder (3).

We describe here the clinical, immunological, and molecular findings of MHC class II deficiency in five patients with novel mutations for the first time from India.

## Materials and Methods

### Patients and Samples

Patients (*n* = 5) diagnosed with MHC class II deficiency at National Institute of Immunohaematology (NIIH) between 2011 and 2017 were included. Informed consent for participating in the study was procured from the family members in accordance with the declaration of Helsinki, and 3 mL peripheral blood was collected in EDTA vacutainers. The study was approved by the Institutional Ethics Committee of NIIH.

Phenotypic prenatal diagnosis (PND) was also provided to one of the affected families. Fetal cord blood (FB) sample (1–2 mL, <0.5% of expected weight in all cases) was collected at 18 weeks of gestation by ultrasound-guided cordocentesis after procuring informed consent from the parents. The FB sample accepted for analysis had a high MCV value (>110 fL) with narrow and single red cell distribution curve. The testing was performed within 3 h of sampling. Maternal contamination was ruled out by Kleihauer–Betke staining and analysis of the variable number of tandem repeats using the *apolipoprotein B*, ACTB2, D1S80, and *IgJH* genes.

### Immunological Workup

Initial investigations involved a complete blood cell count on a Sysmex XS-800i (Sysmex Co., Cobe, Japan) five-part automated hematological analyzer, and lymphocyte subset analysis by flow cytometry using BD Multitest 6-color TBNK reagent followed by acquisition of cells on FACS Aria I; analysis was performed on FACS Diva and FlowJo software (BD Biosciences, San Jose, CA, USA). Serum immunoglobulin levels were estimated by nephelometry (BNProspec, Siemens).

Low absolute CD4 counts prompted flow cytometric evaluation of HLA-DR expression on lymphocytes and monocytes using cell surface markers specific for T cells (anti-CD3 peridinin–chlorophyll–protein Complex:CY5.5 Conjugate), B cells [anti-CD19 allophycocyanin (APC)], monocytes [anti-CD14 phycoerythrin (PE)], and HLA-DR (anti-HLA-DR fluorescein isothiocyanate). The FB sample was also evaluated for HLA-DR expression by the same method (8).

The percentage of naive T cell subsets on CD4^+^ and CD8^+^ cells was measured by flow cytometry using anti-CD45RA PE and anti-CD62L APC procured from BD Biosciences, San Jose, CA, USA.

Clonality of the TCR was assessed by flow cytometric evaluation of TCR-Vβ repertoire by using the IOTest^®^ Beta Mark.

T cell receptor excision circles (TRECs) were measured by an in-house modification of a previously described method (9).

### Molecular Investigations

Molecular investigations in these patients were done by targeted gene capture using a custom capture kit by Medgenome Labs Pvt Ltd., India. The libraries were sequenced on Illumina sequencing platform (mean coverage, >80–100×). The identified mutations were confirmed by Sanger sequencing.

## Results

### Clinical Findings

Recurrent lower respiratory tract infection was the predominant clinical manifestation among the patients. The median age of diagnosis was 5.5 months (range, 3–7 months). One patient (P2) was referred at 3 months of age further to a family history of previous sibling death and was asymptomatic at the time of referral. Infection by opportunistic microorganisms like *Burkholderia cepacia* (P1), *Candida albicans* (P3 and P5), and *Chryseobacterium indologenes* (P4) raised a clinical suspicion for PID in these patients. All patients except P2 had failure to thrive. One of the five patients (P5) belonged to consanguineous parents. The detailed clinical findings are presented in Table 1.

**Table 1:** Clinical findings.

Patient no.	P1	P2	P3	P4	P5
Sex	Male	Male	Female	Male	Male
Age at diagnosis	7 months	3 months	6 months	7 months	5 months
Consanguinity	−	−	−	−	+
Family history	+	+	−	−	+
Age of onset	6 months	NA	3 months	4 months	15 days of life
Failure to thrive	+	−	+	+	+
Bronchopneumonia	+	−	+	−	+
Oral candidiasis	−	−	−	−	+
Organism isolated (source)	*Burkholderia cepacia* (blood culture)	–	*Candida albicans* (bronchoalveolar lavage)	*Chryseobacterium indologenes* (blood culture)	*Candida albicans* (bronchoalveolar lavage)
Other manifestations	Erythematous amoeboid blanching papular Skin rash	Asymptomatic	Delayed milestones, hepatosplenomegaly	Acute respiratory distress syndrome	Chronic diarrhea, nystagmus
Status	Dead	HSCT/dead	HSCT/dead	Alive/on IVIg prophylaxis	Dead
Age at death, cause of death	7 months, severe respiratory failure	4 months, diarrhea, and Gram-negative sepsis	8 months, systemic candidiasis and lung damage	NA	6 months, severe respiratory failure

*HSCT, hematopoietic stem cell transplantation; NA, not applicable*.

### Immunological Findings

**Figure 1:**
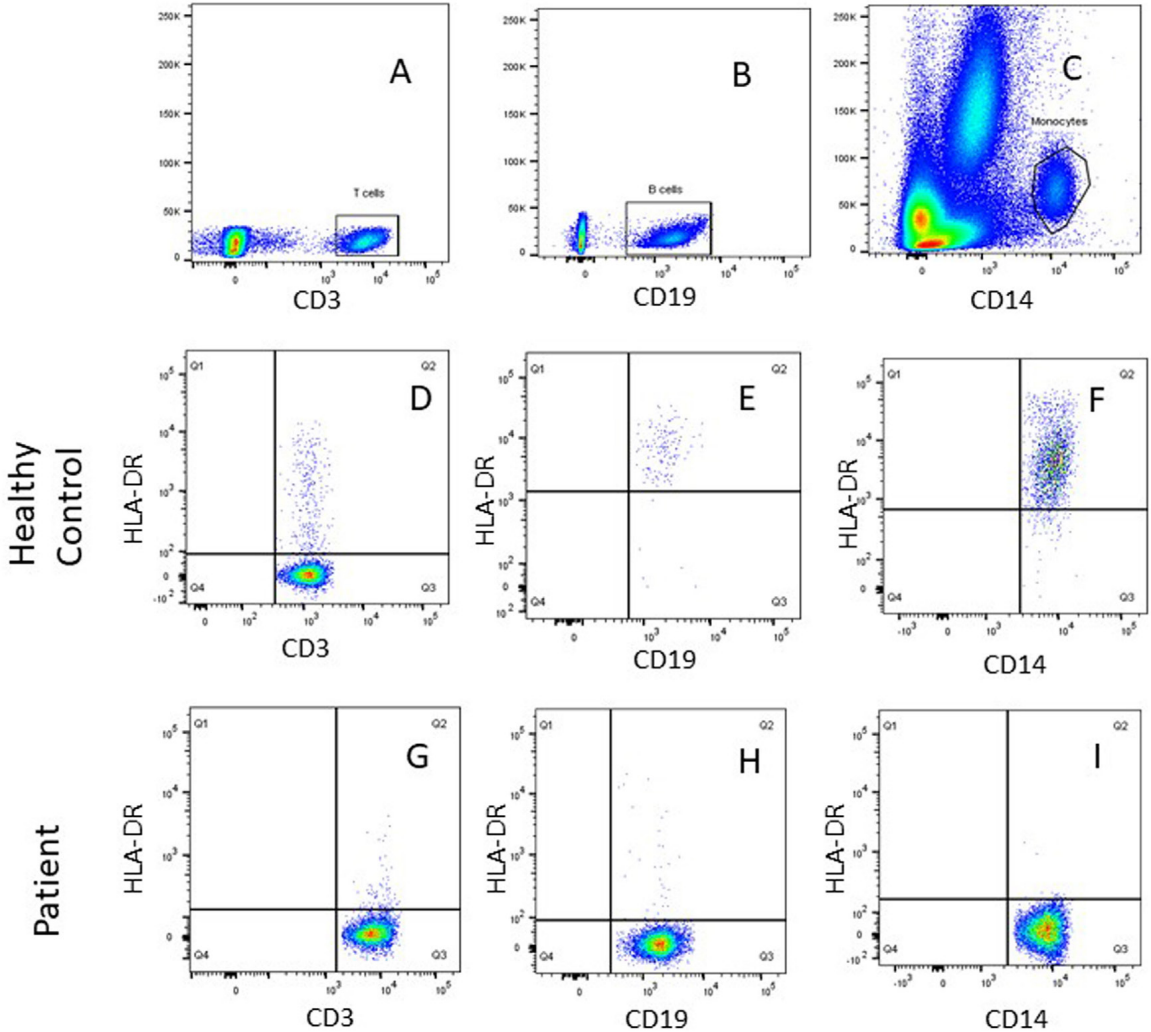
Representative flow cytometric plots showing gating of **(A)** SSC/CD3^+^ T cells, **(B)** SSC/CD19^+^ B cells, and **(C)** SSC/CD14^+^ monocytes using FlowJo software. Dot plot analysis of human leukocyte antigen-antigen D related (HLA-DR) on immune cells showed normal HLA-DR expression for a healthy control on **(D)** T (CD3^+^) cells, **(E)** B (CD19^+^) cells, **(F)** monocytes (CD14^+^), and lack of HLA-DR expression was noted for patient P1on **(G)** T (CD3^+^) cells, **(H)** B (CD19^+^) cells, and **(I)** monocytes (CD14^+^).

**Figure 2:**
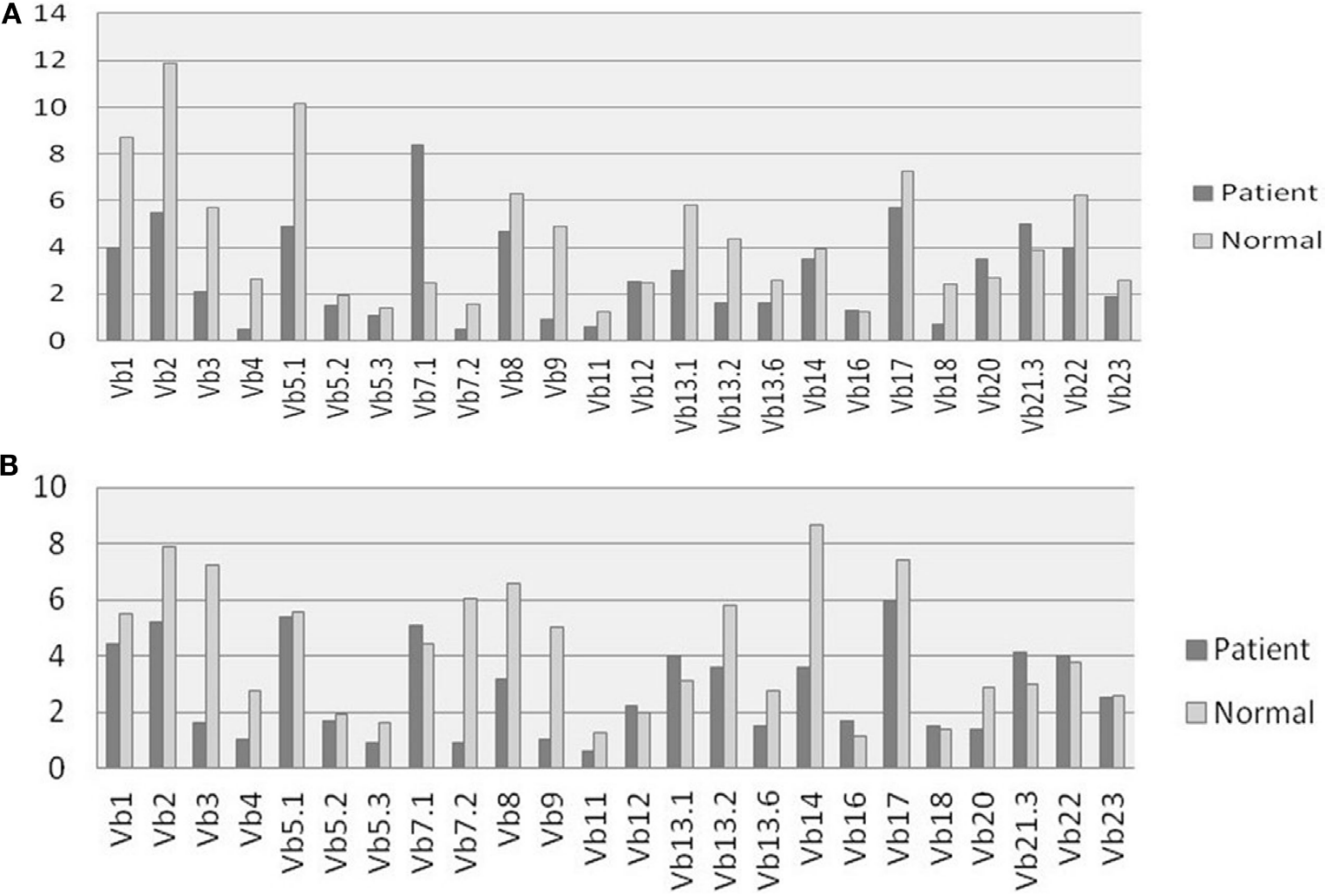
T cell receptor (TCR) Vβ repertoire analysis: comparison of 24 different TCR Vβ families in patient P2 and Healthy control on **(A)** CD4^+^ Th cells and **(B)** CD8^+^ Tc cells.

All patients had an absolute lymphocyte count of >2,500 count/mm^3^, but the absolute CD4^+^ Th cell counts were low (range, 214–685 count/mm^3^) with an inverse CD4:CD8 ratio. The B cell numbers were elevated in one patient (P4) and within normal ranges in other patients. The HLA-DR expression on B lymphocytes and monocytes was absent in all the patients (Figure 1). Serum immunoglobulin levels measured in three patients (P3, P4, and P5) revealed reduced concentrations of IgG, IgA, and IgM consistent with lack of helper T cell-dependent antibody production. Flow cytometric evaluation of TCR-Vβ repertoire on CD4^+^ Th cells and CD8^+^ Tc cells was performed in two patients (P2 and P3). A clonal expansion of TCR Vβ 7.1 on CD4^+^ T cells with a normal distribution of TCR-Vβ repertoire on CD8^+^ T cells (Figure 2) was observed in patient P2. Patient P3 had a normal polyclonal distribution of TCR-Vβ on both CD4^+^ T cells and CD8^+^ T cells. The percentage of naive Th and Tc cells measured in three patients (P2, P4, and P5) revealed a decrease in CD4^+^/CD45RA^+^/CD62L^+^ cells in all the three patients and a normal percentage of CD8^+^/CD45RA^+^/CD62L^+^ cells in two patients (P4 and P5).

Detectable TREC levels were measured in the different patient samples and were within a range of 65–154 copies/reaction. The TREC levels were >100 copies/reaction in the healthy control sample processed along with the patient sample. The detailed immunological findings are presented in Table 2.

**Table 2:** Immunological findings (with age-matched normal ranges).

Patient	P1	P2	P3	P4	P5
Absolute lymphocyte count/mm^3a^	2,860 (3,400–7,600)	3,078 (3,900–9,000)	3,565 (3,400–7,600)	6,715 (3,400–7,600)	3,603 (3,900–9,000)
Absolute T lymphocyte/mm^3a^	1,087 (1,900–5,900)	1,477 (2,500–4,600)	927 (1,900–5,900)	1,544 (1,900–5,900)	1,585 (2,500–4,600)
Absolute Th lymphocyte/mm^3a^	243 (1,400–4,300)	339 (1,800–4,000)	214 (1,400–4,300)	537 (1,400–4,300)	685 (1,800–4,000)
Absolute Tc lymphocyte/mm^3a^	839 (500–1,700)	1,077 (590–1,600)	570 (500–1,700)	873 (500–1,700)	685 (590–1,600)
Absolute B lymphocyte/mm^3a^	1,687 (610–2,600)	1,477 (430–3,000)	1,283 (610–2,600)	5,036 (610–2,600)	1,549 (430–3,000)
HLA-DR expression on monocytes, B cells^a^	Absent	Absent	Absent	Absent	Absent
CD4^+^CD45RA^+^/CD62L^+a^ (%)	ND	42 (64–92)	ND	48 (58–91)	29 (64–92)
CD8^+^CD45RA^+^/CD62L^+a^ (%)	ND	23 (53–88)	ND	82 (47–87)	90 (53–88)
IgG (g/L)	ND	ND	1 (4–15.9)	0.8 (3.5–16.2)	0.86 (3.5–16.2)
IgA (g/L)	ND	ND	1 (0.01–0.91)	<0.23 (0.01–0.91)	<0.23 (0.01–0.91)
IgM (g/L)	ND	ND	0 (0.34–2.06)	0.185 (0.30–1.83)	0.066 (0.30–1.83)
IgE (g/L)	ND	ND	4 (3–423)	<4.45 (3–423)	–
TREC copies/reaction^b^	74	89	65	112	154

*HLA-DR, human leukocyte antigen-antigen D related; IgA, immunoglobulin A; IgE, immunoglobulin E; IgG, immunoglobulin G; IgM, immunoglobulin M; ND, no data; Th, T helper cell; TREC, T cell receptor excision circle*.

*^a^Normal ranges Ref. (10)*.

*^b^TREC copies in healthy control >100 copies/reaction*.

### Genetic Findings

In total, three of five patients harbored a novel mutation in *RFXAP* gene of complementation group D. Patients P1 and P2 presented with a novel homozygous single base pair insertion in exon 1 of the *RFXAP* gene (c.460_461insC), which resulted in a frameshift and premature truncation of protein 21 amino acids downstream to codon 155 (p. Lys155GlnfsTer21). Patient P5 carried a novel homozygous 3′ splice site variation in intron 2 (c.709-1G>T), which affected the invariant AG acceptor splice site of exon 3.

**Figure 3:**
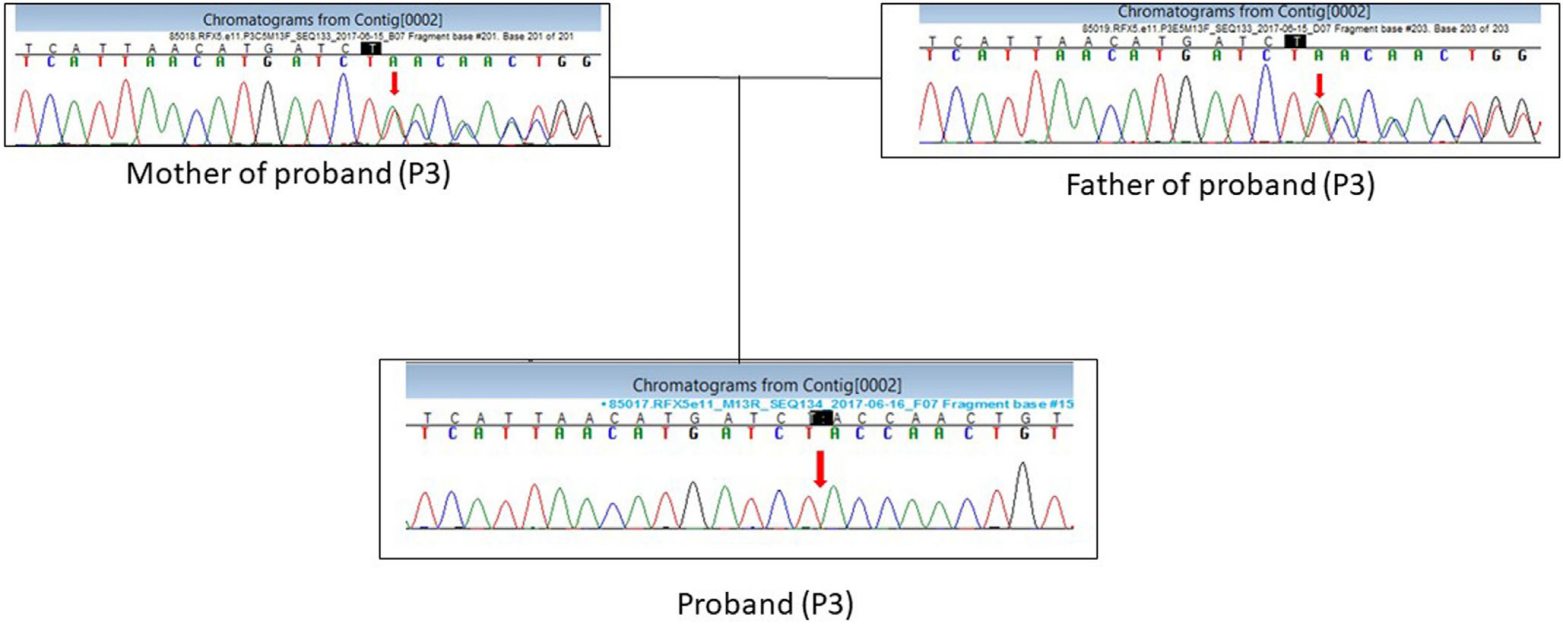
DNA sequence chromatogram showing the c.1154delT mutation in *RFX5* gene in the proband (P3) in a homozygous state and parents in a heterozygous state.

Patient P3 presented with a homozygous single-base pair deletion in exon 11 of the *RFX5* gene (c.1154delT) of complementation group C, which resulted in a frameshift and premature truncation of the protein 33 amino acids downstream to codon 385 (p. Leu385TyrfsTer33; Figure 3).

Patient P4 presented with a novel homozygous nonsense variation in exon 11 of the *CIITA* gene (c.2436 C>A) of complementation group A, which resulted in a stop codon and premature truncation of the protein at codon 812 (p. Cys812Ter).

The *in silico* analysis of these variants was predicted to be damaging by Mutation Taster2 (11). Details of genetic diagnosis are presented in Table 3.

**Table 3:** Genetic findings.

Patient	P1	P2	P3	P4	P5
Gene	*RFXAP*	*RFXAP*	*RFX5*	*CIITA*	*RFXAP*
Exon	Exon 1	Exon 1	Exon 11	Exon 11	Intron 2
Mutation	c.460_461insC (p.Lys155GlnfsTer21)	c.460_461insC (p.Lys155GlnfsTer21)	c.1154delT (p. Leu385TyrfsTer33)	c.2436C>A (p. Cys812Ter)	c.709-1G>T (3′ splice site)
Zygosity	Homozygous	Homozygous	Homozygous	Homozygous	Homozygous
Novelty	Novel	Novel	Novel	Novel	Novel

### Treatment and Outcome

Two patients (P1 and P5) died before HSCT due to severe respiratory failure. Patients P2 and P3 underwent HSCT using myeloablative conditioning (treosulfan, cyclophosphamide, antithymocyte globulin); however, they had a poor survival outcome. Patient P2 who underwent an umbilical cord blood transplant from an unrelated donor died of diarrhea and gram-negative sepsis within 8 days of transplant procedure. Patient P3 underwent HSCT from an HLA identical sibling died post-HSCT due to lung damage and systemic candidiasis. At the time of last available report, patient P4 was on intravenous immunoglobulin prophylaxis and was awaiting transplant.

## Discussion

Major histocompatibility complex class II deficiency is reported majorly in the Mediterranean region and is prevalent in areas with high consanguinity. To the best of our knowledge, this is the first report of MHC class II deficiency from India. Our patients presented clinical symptoms within their first year of life indicating the severity of this form of PID. Recurrent lower respiratory tract infection was the predominant clinical presentation.

In this study, patients displayed a reduced absolute CD4^+^ Th cell count and absent HLA-DR expression on B lymphocytes and monocytes. Notably, these patients presented with normal or elevated absolute B lymphocyte count that could potentially lead to misdiagnosis of MHC class II deficiency as T^−^B^+^ form of severe combined immunodeficiency (SCID). Thus, diagnosis of MHC class II deficiency may be confounded unless HLA-DR expression is evaluated on the immune cells.

Major histocompatibility complex class II deficiency is caused by mutation in genes (*CIITA, RFXANK, RFX5*, and *RFXAP*) that encode any one of the four regulatory factors that control the transcription of MHC class II genes. *RFXANK* gene mutation that accounts for more than two-thirds of all reported cases (1) was not identified in our series. Three of our patients had *RFXAP* gene defect. The RFXAP protein consists of three regions, which are rich in acidic amino acids (DE), glutamine (Q), or basic amino acids (RK) reminiscent of a nuclear localization signal (12). Till date, only five different *RFXAP* gene mutations have been reported (2) with most of the mutations affecting the nuclear localization signal or DE regions. The *RFXAP* gene defect in exon 1 of our patients P1 and P2 also affects the nuclear localization signal and the mutation in intron 2 of patient P5 is expected to affect the formation of C-terminal domain, which is required for binding to the RFX complex (13).

Fifth member of the RFX has a DNA-binding domain (DBD) (7) located in residues 90–166 and 407–614, which binds to the ssDNA of the X-boxes before transcription (2) and is believed to interact with CIITA (14). Of the six *RFX5* gene mutations reported in the literature, five mutations result in a premature stop codon before the second DBD sequence (2). In line with this finding, the mutation in Exon 11 of *RFX5* of our patient (P3) also leads to the premature truncation of the protein by the creation of a premature stop codon just before the second DBD and could potentially be the underlying cause of MHC class II deficiency.

The expression of CIITA is essential for the transcription of all MHC class II genes (15). Till date, 12 different mutations have been reported in the *CIITA* gene, which are either missense or nonsense mutations (2). Patient P4 described in this study harbored a novel nonsense mutation in the *CIITA* gene, which affects the transcription of MHC class II genes.

Despite such genetic heterogeneity, these patients had a similar clinical presentation. Traditional Sanger sequencing of individual genes is time consuming in such cases and delays the diagnosis. The strategies that are usually employed for genetic diagnosis of MHC class II deficiency are founder mutation screening and study of polymorphic markers that flank the MHC class II genes in case of consanguinity. Another approach involves a functional test to identify the gene defect, which is based on the direct correction of the genetic defect by transduction of cells from affected individuals with lentiviral vectors encoding CIITA, RFXANK, RFX5, or RFXAP to diagnose and classify MHC class II-deficient patients (16). Currently, with the application of next-generation sequencing (NGS) technology, screening of all the four causative factors can be performed at the same time, thereby reducing the cost and time for getting a diagnosis. Thus, we opted for NGS for genetic confirmation of MHC class II deficiency in our patients.

Hematopoietic stem cell transplantation is the only known curative treatment for MHC class II expression deficiency. However, the success rate is reported to be poor in MHC class II-deficient patients compared to other forms of SCID (17). This is attributed to severe infections, multiorgan failure, and acute graft-versus-host disease observed in relation to pre-existing (pre-HSCT) viral infections (18). As stated in a recent report, the reason for low survival rate in these patients may be due to the presentation of donor antigens by donor antigen-presenting cells to recipient T cells leading to graft rejection (19). Even with immunosuppression, residual host immunity in these patients is reported to be adequate to cause graft rejection. A need for a second transplant was observed in multiple reports of these patients (19). Our data support this trait, where a poor survival outcome was observed in our patients (P2 and P3). Although patient P2 had a presymptomatic diagnosis and received an early transplant, the patient expired within 8 days of transplant due to severe sepsis. For this reason, PND is highly useful in families affected with MHC class II deficiency. As the decision for termination of pregnancy is based on the test results, the preferred procedure for PND is genetic confirmation in the index case and parents and then performing PND by chorionic villus sampling or amniocentesis. However, in cases where a molecular diagnosis is not available in the index case at the time of PND, families can opt for phenotypic PND on cordocentesis sample at 18 weeks of gestation by flow cytometry. Although cordocentesis is done at a later stage of pregnancy (16–18 weeks), the results are usually available within 24 h of the procedure thus providing sufficient time to the family for safe termination of pregnancy (i.e., up to 20th week of gestation). One of our affected families (Patient P1) approached us for PND at a late gestational age of pregnancy. As genetic diagnosis was not available in the index case at the time of PND and we were limited by time for performing the molecular study, phenotypic PND on cordocentesis sample was provided to the family. The fetus was found to be affected with MHC class II deficiency and was eventually terminated (8).

Detectable TREC levels (range, 65–154 copies/reaction) were measured in all the patients. TREC-based newborn screening (NBS) has proven to be extremely beneficial in picking up classical forms of SCID (20); however, TREC levels in MHC class II patients have been reported to be higher than the NBS TREC cut-off for SCID and other T cell lymphopenia conditions (21, 22). Our study also supports this finding and highlights that MHC class II deficiency may be missed by TREC-based NBS programs.

## Ethics Statement

This study was carried out in accordance with the recommendations of Institutional Ethics Committee of National institute of Immunohaematology for research on human Subjects in accordance with the Declaration of Helsinki. All subjects gave written informed consent in accordance with the Declaration of Helsinki. The protocol was approved by the Institutional Ethics Committee of National institute of Immunohaematology.

## Author Contributions

JA analyzed the data and wrote the manuscript. MG, AD, SM, MK, and GH were involved in performing laboratory investigations of the different cases. MD and NS supervised the clinical care of the patients. PT helped in the collection of samples and the clinical details. RV provided the genetic data. MM supervised the study and reviewed the manuscript.

## Conflict of Interest Statement

The authors declare that the research was conducted in the absence of any commercial or financial relationships that could be construed as a potential conflict of interest.
